# Exploration of the genetic influence of MYOT and MB genes on the plumage coloration of Muscovy ducks

**DOI:** 10.1515/biol-2022-0836

**Published:** 2024-04-01

**Authors:** Guo-Bo Sun, Yan-Feng Lu, Xiu-Jun Duan

**Affiliations:** College of Animal Science and Technology, Jiangsu Agri-animal Husbandry Vocational College, No. 8 of Fenghuang East Road, Hailing District, Taizhou City, Jiangsu Province, 225300, China

**Keywords:** MB, Muscovy duck, MYOT, plumage color, RT-PCR, western blotting

## Abstract

Plumage color, a pivotal attribute delineating diverse Muscovy duck strains, assumes considerable significance within the field of Muscovy duck breeding research. This study extends the existing research by delving into the hereditary aspects of genes associated with plumage coloration in Muscovy ducks. The principal objective is to discern marker genes conducive to targeted breeding strategies based on plumage color, thereby furnishing indispensable technical foundations for the development of novel Muscovy duck varieties. Our investigation focused on scrutinizing the impact of MYOT and MB genes on the genetic expression of plumage color at both the RNA and protein levels in Muscovy ducks. The results elucidate that black Muscovy ducks manifest markedly elevated mRNA and protein expression levels of MYOT and MB genes in comparison to their white counterparts, indicating that both genes may play a constructive regulatory role in the context of plumage coloration in Muscovy ducks. The outcomes of this study delineate a discernible correlation between MYOT and MB genes and the plumage coloration in Muscovy ducks. Employing gene expression analysis, we successfully identified candidate genes that may be intricately linked to the determination of plumage color in these ducks.

## 1 Introduction

Originating from the tropical regions of South and Central America, Muscovy ducks are primarily bred for their meat, owing to their substantial size, rapid growth, ease of nutritional management, elevated proportion of lean meat, and appealing flavor [1]. In China, the breeding history of these ducks spans over three centuries, initially under free-range conditions, but presently, they are predominantly reared in extensive poultry farming facilities. Currently, Muscovy ducks are classified according to their visual characteristics and plumage colors, and are classified as black Muscovy ducks, white Muscovy ducks, and mixed-color Muscovy ducks [2].

White Muscovy ducks are predominantly characterized by a white plumage, with some ducks exhibiting a minimal presence of fine black feathers on their heads. Notably, their bills are pink, adorned with dark red caruncles at the base. The plumage displays an iridescent light gray hue, further accentuated by orange-yellow legs and webbed feet. Conversely, black Muscovy ducks exhibit a dark blackish coloration with a distinctive dark green luster. Typically, there are occasional white primary and secondary feathers on their wings. Their bills are characterized by a red hue with discernible black spots, accompanied by dark red caruncles at the base. In contrast, mixed-colored Muscovy ducks showcase a diverse array of plumage, spanning from black to white, featuring black on the back and distinct white patches on the lower neck and primary feathers of the wings [2,3].

Plumage colors in duck breeds contribute significantly to their economic valuation. This is particularly noteworthy in the case of the black Muscovy duck, where plumage color holds pronounced importance due to its correlation with superior meat quality as compared to its white counterpart. The preference for premium duck meat among Chinese consumers enhances the desirability of the black Muscovy duck, underscoring the economic significance associated with specific plumage traits. Nevertheless, the genetic transmission of plumage color in Muscovy ducks presents a challenge characterized by its variability across generations and disparate physiological stages. Conventional breeding approaches have encountered limitations in establishing consistent inheritance patterns for plumage color. This underscores the imperative for immediate research endeavors to unravel the genetic foundations underlying the plumage coloration specifically observed in black Muscovy ducks.

Previous studies have demonstrated that the plumage color in poultry is a complex trait governed by a multitude of genes [3,4]. The regulation of this trait involves intricate relationships, complex gene–gene interactions such as dominance and epistasis. Consequently, the precise control of plumage color in Muscovy duck offspring poses a considerable challenge due to the intricate nature of these genetic interactions. Notwithstanding the complexity of this phenomenon, there has been a notable lack of emphasis on comprehending the genetic mechanisms and molecular expressions linked to Muscovy duck plumage color. Specifically, research in the domain of gene transcriptomics pertaining to Muscovy duck plumage color remains substantially unexplored.

The MYOT gene encodes a protein known as myotilin, which assumes a critical role in preserving the structure and stability of muscle cells. This protein actively participates in the processes of muscle cell contraction and relaxation, thereby contributing to the mechanical stability of muscle fibers. Myotilin is an indispensable constituent of muscle fiber tissues, playing a pivotal role in maintaining their structural integrity. Mutations in the MYOT gene have been implicated in a range of muscle-related disorders. Conversely, the MB gene encodes myoglobin, a protein inherent to muscle tissues. MB serves as a molecule for binding and storing oxygen within muscle cells, playing a crucial role in oxygen storage and release during high-intensity activities, thereby supporting the energy requirements of muscles. The expression patterns of these genes may be linked to distinct feather color traits [5,6].

In this study, both white and black Muscovy ducks were selected as subjects, facilitating a comprehensive examination of plumage color genetics. Tissue samples were systematically collected, and subsequent extraction procedures yielded both RNA and protein. Polymerase chain reaction (PCR) technology was utilized to analyze the mRNA expression of MYOT and MB genes across diverse Muscovy ducks. Furthermore, western blotting experiments were conducted to assess the expression levels of MYOT and MB genes in these ducks. This comprehensive approach allowed for the establishment of a preliminary regulatory network for plumage color traits in black Muscovy ducks, thereby laying a valuable foundation for future breeding initiatives aimed at improving black Muscovy duck populations.

## 2 Materials and methods

### 2.1 Research materials

#### 2.1.1 Preparation of experimental ducks

Over the course of the brooding period extending to the conclusion of the initial laying phase (at 350 days of age), a total of 100 robust Muscovy ducks, encompassing both white and black varieties and demonstrating uniform traits, were deliberately chosen and raised. Throughout the brooding period, they were raised on land, and subsequently, during the rearing phase, they were accommodated in cages and raised collectively in groups. A total of 50 adult Muscovy ducks with black feathers and 50 with white feathers were carefully chosen for the study. From each feather color group, ten representative ducks were further selected. In order to achieve consistency in analysis and reduce inter-sample errors, we mixed the RNA of nine Muscovy ducks with similar phenotypes in three equal parts, forming three new samples. Tissue samples, including hair follicles from the chest and legs, as well as muscle tissue surrounding the hair follicles, were collected from each individual Muscovy duck. Additionally, hair follicle tissue samples from wing feathers were collected and stored at −80°C. The former set of samples was utilized for mRNA expression analysis, while the latter was employed in western blotting experiments.


**Ethical approval:** The research related to animal use has been complied with all the relevant national regulations and institutional policies for the care and use of animals, and has been approved by the Ethics Committee of Jiangsu Agri-animal Husbandry Vocational College.

#### 2.1.2 Main instruments and reagents

The experimental procedures involved the utilization of various instruments and reagents, including Trizol reagent, RIPA lysis buffer, BCA reagent, a centrifuge, a homogenizer, agarose gel electrophoresis equipment, a nanophotometer, a PCR amplification instrument, polyacrylamide gel electrophoresis equipment, DEPC water, filter paper, and a 0.5% ponceau S solution.

### 2.2 Research methods

At the ages of 120 days and 300 days, feather follicle tissue samples were collected from two distinct Muscovy duck varieties – white and black – for an in-depth examination of their plumage color genetics. The collection process targeted specific regions of the feather follicle tissue that exhibited unique characteristics in plumage color transformation, predominantly showcasing the transition from black to white. Following collection, the tissue samples underwent processing to extract RNA and protein in adherence to relevant protocols.

#### 2.2.1 Sample RNA extraction and reverse transcription reaction

Trizol reagent was utilized for the extraction of RNA from feather follicles obtained from both black and white Muscovy ducks. The feather follicle tissue was collected and then finely ground using liquid nitrogen. Following this, the ground tissue underwent a 10 min treatment with Trizol reagent to induce lysis. Chloroform was introduced into the mixture, followed by a 15 s shaking period and subsequent settling at room temperature for approximately 2–3 min. Following a 15 min centrifugation step, the solution underwent phase separation into three distinct layers. The RNA, found in the supernatant, was meticulously extracted and transferred to a clean EP tube. Subsequently, isopropanol was added to the extracted RNA, and the mixture was allowed to stand for 10 min before undergoing an additional 10 min centrifugation step. After discarding the supernatant, the remaining RNA precipitate was washed with 75% alcohol to eliminate any residual organic reagents. Subsequently, the mixture was centrifuged for 5 min. Finally, the concentration of RNA was determined by adding DEPC-treated water.

The RNA content was assessed using agarose gel electrophoresis, and the purity and integrity of the RNA were examined using a nanophotometer.

To initiate the reverse transcription reaction, total RNA extracted from the feather follicle tissues of both black and white Muscovy ducks served as templates. The reaction protocol involved mixing 2 μL of LOligo dT (18) (50 μM), 4 μL of RNA (2 μg), and 12 μL of nuclease-free double distilled water, followed by incubation at 65°C for 5 min and subsequent cooling in an ice bath for 5 min. Subsequently, 1 μL of LRNase inhibitor (40 u/µL), 4 μL of 5× reaction buffer, 2 μL of dNTPs (10 mM), and 1 μL of LM-MuLV were sequentially added to synthesize cRNA for long-term storage.

#### 2.2.2 Primer sequence design

The PCR primer sequences were devised utilizing the published GenBank data for the MYOT and MB gene sequences of ducks (Table 1).

**Table 1:** PCR primer sequences

Gene	Gene primer sequence (5′→3′)	
MYOT	F:TGACACTGGTGTAGGCAGGA	R:TGGATTTTCCCTACAGCCTAGAAG
MB	F:GCCATAGGCAGCACTTGAGA	R:GGTGGTCATGGAAAAGTCTCATC
β-actin	F:CTCTGACTGACCGCGTTACT	R:TACCAACCATCACACCCTGAT

#### 2.2.3 RT-PCR amplification of MYOT and MB genes

The initial first-strand cDNA, subject to a ten-fold dilution, was utilized as the template for the design of specific primers for subsequent PCR amplification. The PCR reaction was composed of the following components: 10 μL of 2× real-time PCR master mix (containing SYBR Green), 1 μL of cRNA, 2 μL of primer mix (F/R at 10 μM concentration each), 7 μL of 0.1% DEPC water, resulting in a total reaction volume of 20 μL.

The PCR reaction was executed with the following program: pre-denaturation at 95°C for 5 min, denaturation at 95°C for 15 s, annealing at 60°C for 20 s, and extension at 72°C for 1 min; this cycle was repeated 40 times. Subsequent to the cycles, a final extension step was conducted at 72°C for 8 min, and the reaction was then stored at 4°C.

#### 2.2.4 Protein extraction and concentration determination of samples

The feather follicles from both black and white Muscovy ducks underwent meticulous weighing and were subsequently combined with RIPA lysis buffer containing 1% phenylmethylsulfonylfluoride. The resultant mixture was homogenized for 2–3 min to achieve comprehensive blending, followed by centrifugation at 4°C for 15 min to facilitate component separation. The resulting supernatant was carefully transferred to a new EP tube for subsequent utilization.

A sequential set of procedures was undertaken for protein concentration detection through a BCA assay. Initially, a protein standard was prepared by diluting it from a 5 to a 0.5 mg/mL working solution using RIPA buffer (Table 2). Specifically, 10 μL of the diluted standard was extracted, and the volume was then increased to 100 μL (typically preparing two copies). Furthermore, a BCA working solution was created by combining BCA kit solution A with solution B in a 50:1 ratio, and the total volume needed for each well (200 μL) was calculated. In the process of protein concentration detection and sample loading, 4 μL of the protein sample was introduced into each well, and each well received an additional 16 μL of RIPA, yielding a total volume of 20 μL. This resulted in a fivefold dilution of the protein sample, corresponding to a final protein concentration multiplied by 5. Following this, 200 μL of the working BCA solution was added to each well. Subsequently, the samples underwent incubation at 37°C for a duration of 30 min, during which the protein concentration was monitored using suitable equipment. Prior to loading, careful and thorough mixing of the samples was guaranteed to uphold consistency and accuracy throughout the experiment.

**Table 2:** RIPA dilution standard

96-well plate standard amount (μL)	0	1	2	4	8	12	16	20
RIPA (or PBS) (μL)	20	19	18	16	12	8	4	0
The final concentration of the standard (mg/mL)	0	0.025	0.05	0.1	0.2	0.3	0.4	0.5

Note: Repeat 3 wells when loading sample.

#### 2.2.5 Western blotting

Western blotting is a highly precise and sensitive technique employed for the identification of protein expression. It comprises three primary steps: SDS-PAGE, electrotransfer, and hybridization. Initially, the protein samples were subjected to separation based on their molecular weight through polyacrylamide electrophoresis. Subsequently, they were transferred onto a hybrid membrane, commonly referred to as a blot. In the concluding phase, the target protein was selectively identified employing a complex comprising primary and secondary antibodies. The protein gel obtained from polyacrylamide gel electrophoresis was utilized in this process. Filter paper, tailored to match the gel size, was immersed in transfer electrophoresis buffer and placed on the Scotch-Brite Pad. Following the careful elimination of bubbles, a damp filter paper was placed on the negative end of the gel, whereas a moist nitrocellulose membrane of equivalent size was positioned on the anode surface of the gel. The discharge of bubbles was meticulously executed to ensure precise placement. Another filter paper was then positioned on the positive end of the membrane, and the process was again followed by bubble discharge. The entirety of the setup was enclosed with the Scotch-Brite Pad.

Subsequently, the assembled sample “sandwich” was positioned at the center of a plastic support and introduced into the electric transfer device. Electric transfer buffer was introduced, facilitating the transfer of proteins from the gel to the nitrocellulose membrane under a voltage of 14 V, maintained at 4°C for a duration of 4 h or overnight.

Following the electrotransfer, the filter membrane was immersed in a 0.5% ponceau S solution for 5 min to visualize the protein bands. Subsequently, the protein underwent a decolorization process in water for 2 min, during which photographs were taken. For the determination of molecular weights, Indian ink was employed to stain the molecular weight standard, followed by thorough decolorization in water.

The filter membrane was enclosed in a plastic bag, and each piece of paper was treated with 5 mL of blocking buffer to hinder specific antibody binding. Following an hour of shaking at room temperature, the buffer was decanted. The primary antibody was diluted in the blocking buffer and allowed to incubate at room temperature for 1 h. Subsequently, the filter membrane was moved to a plastic container and subjected to four washes with 200 mL of PBS, employing agitation during each wash.

The aforementioned procedure was repeated for the dilution of the horseradish peroxidase-labeled secondary antibody in blocking buffer. Subsequently, the filter membrane was submerged in 100 mL of freshly prepared 3,3′-diaminobenzidine substrate solution, leading to color development in approximately 2–3 min. The reaction was halted by rinsing with water, and photographs were taken to document the results.

### 2.3 Statistical analysis

The data derived from our experiments underwent comprehensive statistical analysis using appropriate software, such as SPSS. To ascertain the significance of differences in gene expression levels between black and white Muscovy ducks, an independent sample *t*-test was executed where applicable. The predetermined threshold for statistical significance was established at *p* < 0.05.

## 3 Results

### 3.1 mRNA expression results

**Figure 1:**
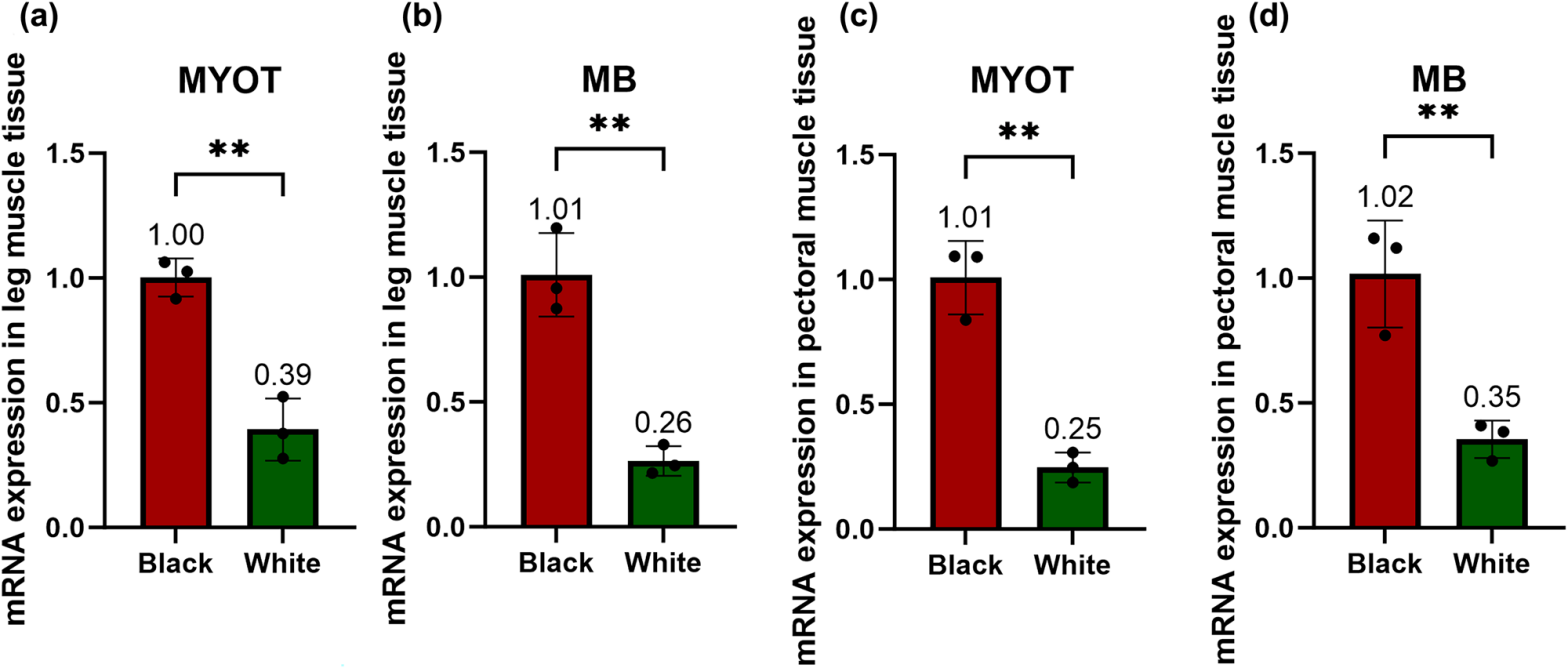
Levels of mRNA expression of the MYOT and MB genes in muscle tissues of black and white Muscovy ducks. Muscovy ducks of different plumage colors. Relative expression of mRNA in pectoral muscle tissue. Relative expression of mRNA in leg muscle tissue.

Our investigation involved the scrutiny of mRNA expression in muscle tissues from both black and white Muscovy ducks, specifically focusing on the MYOT and MB genes. The outcomes indicate significantly elevated mRNA expression levels of MYOT and MB genes in black Muscovy ducks when juxtaposed with their white counterparts. This observation implies a pronounced positive correlation between the expression of MYOT and MB genes and the manifestation of the black feather trait in Muscovy ducks, as illustrated in Figure 1.

### 3.2 Protein expression analysis

In our investigation, we delved into the examination of protein levels to scrutinize the multi-protein expression of MYOT and MB genes. The findings revealed heightened protein expression of MYOT and MB genes in black Muscovy ducks in comparison to their white counterparts. However, it is noteworthy that the differences in protein expression of MYOT and MB genes among Muscovy duck populations exhibiting diverse plumage colors did not reach statistical significance, as indicated in Table 3.

**Table 3:** Outcomes of protein expression for the MYOT and MB genes

Name of gene	Expression quantity	
	Black Muscovy duck	White Muscovy duck
MB	0.91 ± 0.04	0.72 ± 0.03
MYOT	0.92 ± 0.07	0.78 ± 0.09

## 4 Discussion

The MYOT gene consists of ten segments, referred to as exons, collectively encoding a sequence of 498 amino acids. This amino acid sequence is predominantly expressed in skeletal muscles and the heart [7]. Functionally, the gene serves as a positive regulator in muscle tissue, demonstrating clear dominance. In humans, the MYOT gene is situated on 5q31 and is associated with limb-girdle muscle disease [8]. Mutations in the MYOT gene have been identified as causative factors for muscular dystrophy and other related disorders [9].

While there has been limited research on the MYOT gene in poultry, the existing studies have predominantly centered on its correlation with meat quality. Notably, Silva et al. established a robust association between MYOT and its related genes with beef quality [10]. Additionally, Adoligbe et al. uncovered the pivotal role of the MYOT gene in myogenic differentiation, proposing that specific regions within the gene could function as molecular markers for the genetic enhancement of beef [11].

As of the present, there is a paucity of information regarding the involvement of the MYOT gene in regulating plumage color traits in Muscovy ducks within existing research. Notably, MB has been identified as a fundamental factor influencing the color of pork, where the diverse shades of meat color are contingent upon the levels of MB present. The capacity of MB to absorb visible light and serve as a pigment is attributed to the resonance property of its conjugated double bond group. Analogous to the MYOT gene, the MB gene exhibits dominance and plays a pivotal role in actively regulating both meat quality and color [12]. For instance, Liu et al. found that elevated MB expression in pork is typically linked with desirable meat color and reduced water content [13]. Windarsih et al. proposed that MB could serve as an indicator for the assessment and detection of meat quality [14]. Nevertheless, akin to the MYOT gene, the specific relationship between the expression of the MB gene and the plumage color traits of Muscovy ducks has been investigated in a limited number of prior studies. Therefore, in this particular study, our emphasis was on scrutinizing the MYOT and MB genes. PCR analysis was utilized to scrutinize the mRNA expression of these genes in Muscovy ducks displaying diverse plumage colors. Furthermore, western blotting was employed to validate the protein expression of both MYOT and MB genes in Muscovy ducks.

The outcomes of the study imply a connection between the MYOT and MB genes and the inheritance of plumage color in Muscovy ducks. These genes were observed to play a positive regulatory role, exerting a significant influence. It is noteworthy that the protein expression levels could influenced plumage coloration in Muscovy ducks. Various factors, such as the stage of plumage color development, environmental conditions, feeding practices, and nutritional levels, may contribute to diverse outcomes in plumage color breeding within Muscovy ducks of the same breed. The exploration of genetic expression governing plumage color yields valuable insights into the techniques for refining plumage color-based breeding. An understanding of the candidate genes dictating plumage color genetic traits in Muscovy ducks is essential for delving into molecular structures, conducting genetic research, and effectively applying these genetic traits. This knowledge serves as crucial guidance for advancing the field of breeding and genetic research in Muscovy ducks.

Currently, there are few studies that have explored the genetic expression of Muscovy duck plumage color in relation to MYOT and MB genes, highlighting the need for additional research in this area. Furthermore, this study lacks ongoing verification, resulting in an incomplete understanding of the molecular regulation mechanism governing the inheritance of plumage color in black Muscovy ducks, and there are still some deficiencies to be addressed. To address these gaps, we plan to undertake targeted follow-up breeding and validation studies. Acknowledging the challenges posed by limited sample availability and indicators, we recognize the scarcity of studies investigating mRNA and protein expression, which further suffer from a lack of systematic approach. Consequently, it is imperative to conduct additional comparative analyses and ongoing breeding studies of related genes at different levels. This approach aims to efficiently advance the breeding progress through molecular-assisted selection in black Muscovy ducks.

## 5 Conclusion

The findings of this study underscore a distinct association between the MYOT and MB genes and the transmission of plumage color in Muscovy ducks. Building upon these results, we have successfully identified potential genes linked to Muscovy duck plumage color, laying a crucial groundwork for subsequent gene validation and breeding investigations. This discovery holds promise for advancing our understanding and potentially influencing the breeding strategies for plumage color in Muscovy ducks.
